# Sex-Specific Patterns in Blood Pressure and Vascular Parameters: The MUJER-EVA Project

**DOI:** 10.3390/jcdd12050175

**Published:** 2025-05-05

**Authors:** Alicia Saz-Lara, Arturo Martínez-Rodrigo, Eva María Galán-Moya, Irene Martínez-García, Iris Otero-Luis, Carla Geovanna Lever-Megina, Nerea Moreno-Herraiz, Iván Cavero-Redondo

**Affiliations:** 1 CarVasCare Research Group, Faculty of Nursing, University of Castilla-La Mancha, 16071 Cuenca, Spain; alicia.delsaz@uclm.es (A.S.-L.); irene.mgarcia@uclm.es (I.M.-G.); iris.otero@uclm.es (I.O.-L.); carlageovanna.lever@uclm.es (C.G.L.-M.); nerea.moreno@uclm.es (N.M.-H.); ivan.cavero@uclm.es (I.C.-R.); 2 COMETA Research Group, Informatics Systems Department, University of Castilla-La Mancha, 16071 Cuenca, Spain; 3 Cancer Pathophysiology and Therapy Lab, Institute of Biomedicine (IB-UCLM), Universidad de Castilla La Mancha, 02008 Albacete, Spain; evamaria.galan@uclm.es; 4 Physiology and Cell Dynamics Group, Instituto de Investigación Sanitaria de Castilla la Mancha (IDISCAM), Universidad de Castilla La Mancha, 02008 Albacete, Spain; 5 Traslational Oncology Group UCLM-GAI Albacete, Universidad de Castilla-La Mancha-Servicio de Salud de Castilla-La Mancha, 02008 Albacete, Spain; 6 Faculty of Nursing, Universidad de Castilla-La Mancha, 02006 Albacete, Spain

**Keywords:** blood pressure, vascular parameters, sex differences, adults

## Abstract

Recent evidence suggests that sex-related differences in cardiovascular health extend beyond traditional risk factors, affecting vascular structure and function. This study aimed to examine sex differences in vascular parameters, including central and peripheral blood pressure, pulse wave velocity (PWv), augmentation index at 75 bpm (AIx75), cardiac output, stroke volume, and peripheral vascular resistance, using harmonized data from three population-based cohorts (EVasCu, VascuNET, and ExIC-FEp) as part of the MUJER-EVA project. A total of 669 adult participants were included in this pooled cross-sectional analysis. Sex-stratified comparisons were conducted using multiple linear regression models adjusted for anthropometric, sociodemographic, and clinical covariates. The results showed that men had significantly higher values for central and peripheral blood pressure (*p* < 0.001), PWv (*p* = 0.003), cardiac output (*p* < 0.001), and stroke volume (*p* < 0.001), whereas women presented higher values of AIx75 (*p* < 0.001) and peripheral vascular resistance (*p* = 0.002). These differences remained statistically significant after full adjustment for potential confounders. These findings highlight the need to consider sex as a key biological variable in cardiovascular research and clinical decision-making. Incorporating sex-specific reference values and personalized treatment strategies could improve vascular health assessment and the effectiveness of cardiovascular disease prevention.

## 1. Introduction

Cardiovascular diseases (CVDs) remain the leading cause of mortality worldwide, with approximately 17.9 million deaths annually [1]. Among the various clinical and subclinical markers of cardiovascular risk, arterial stiffness and central blood pressure have emerged as independent predictors of adverse cardiovascular outcomes [2,3]. These parameters reflect early vascular aging (EVA) and play a key role in identifying individuals at increased risk, even before the onset of overt clinical disease [4]. Therefore, monitoring vascular function and hemodynamics in the general population is essential to guide prevention strategies and mitigate the long-term cardiovascular burden [5].

In recent years, the importance of incorporating sex and its perspectives into cardiovascular research has been recognized [6]. Biological sex differences, along with sex-related social and behavioral factors, are known to influence cardiovascular risk profiles, access to care, and response to treatment [7,8]. Despite this finding, in most clinical and epidemiological studies, either women are still underrepresented or sex-stratified analyses are not performed [9]. It is crucial to fill these information gaps, as men and women may have different pathophysiological trajectories and risk patterns in relation to vascular aging and CVD development [10].

There is increasing evidence that sex-related differences extend beyond traditional risk factors and into the realm of vascular structure and function [11]. Studies have shown, for example, that women tend to have higher augmentation index values but lower pulse wave velocity (PWv) and blood pressure values than men, indicating different profiles of arterial stiffness and wave reflection [12,13,14]. These disparities may be explained by hormonal influences, differences in arterial size, or vascular tone regulation [15,16] and have implications for the evaluation and management of cardiovascular risk in both sexes.

We hypothesized that sex is an independent determinant of vascular phenotype and that these differences remain significant, even after adjusting for age, body composition, educational level, occupational status, family history of CVD, and smoking. Thus, this study aimed to provide a comprehensive, sex-stratified analysis of vascular function (central and peripheral blood pressure, PWv, augmentation index normalized to 75 beats per minute (AIx75), cardiac output, stroke volume, and peripheral vascular resistance) via harmonized data from diverse population-based cohorts. Additionally, this study aimed to examine whether sex-based differences in these blood pressure and vascular parameters persist after controlling for demographic, anthropometric, and socioeconomic confounders.

## 2. Materials and Methods

### 2.1. Design and Sample Characteristics

This study is part of the MUJER-EVA project, a pooled cross-sectional analysis that integrates data from three different databases: EVasCu [17], VascuNET, and ExIC-FEp [18]. These datasets include adult participants, with the aim of evaluating vascular aging and cardiovascular risk across diverse populations. The current analysis focused on individuals for whom complete blood pressure and vascular data were available. Participants with missing or inconsistent data were excluded following harmonization and quality control procedures. In cases of minimal missing data (<1%), imputation was performed using the median for continuous variables and the mode for categorical variables. The final analytical sample included both male and female participants, allowing for sex-stratified comparisons of cardiovascular parameters.

Participants in the EVasCu cohort were healthy adults (≥18 years) who were clinically stable for at least 6 weeks prior to enrollment, excluding individuals with diagnosed pathologies or those receiving pharmacological treatment. The VascuNET cohort included a heterogeneous population composed of both healthy subjects and individuals with vascular or cardiometabolic pathologies, provided that they were clinically stable and did not participate in other studies. The participants in the ExIC-FEp cohort were sedentary adults aged ≥ 40 years with a confirmed diagnosis of heart failure with preserved ejection fraction (HF-PEF), who were clinically stable for at least 6 weeks and under optimal medical treatment; strict exclusion criteria were applied to rule out other significant cardiac or systemic comorbidities. In all cohorts, individuals with unstable cardiovascular disease or significant health problems that could affect vascular parameters were excluded on the basis of clinical assessment. Medication use (e.g., antihypertensives or statins) was not uniformly recorded across the datasets; however, ExIC-FEp participants were required to have stable drug treatment prior to inclusion.

### 2.2. Ethical Considerations

The studies included in this pooled analysis were conducted in accordance with the ethical standards outlined in the Declaration of Helsinki and the World Health Organization guidelines for observational research. All research protocols were approved by the corresponding local Research Ethics Committees: EVasCu (REG: 2022/PI2022), VascuNET (REG: 2023/PI1823), and ExIC-FEp (REG: 2022/PI2122). All participants provided written informed consent prior to enrollment. Data were anonymized for analysis, and confidentiality was maintained throughout the study in compliance with Spanish data protection laws (Organic Law 3/2018 on Personal Data Protection and the Guarantee of Digital Rights) and the European General Data Protection Regulation (EU Regulation 2016/679 of the European Parliament and of the Council, 27 April 2016).

### 2.3. Variables

#### 2.3.1. Dependent Variables

Cardiovascular parameters were the primary dependent variables in this study. These included central systolic and diastolic blood pressure, peripheral systolic and diastolic blood pressure, central and peripheral pulse pressure, AIx75, PWv, cardiac output, stroke volume, and peripheral vascular resistance.

Central systolic and diastolic blood pressure parameters, central pulse pressure, AIx75, PWv, cardiac output, stroke volume, and peripheral vascular resistance were measured noninvasively via oscillometric techniques with a Mobil-O-Graph device (IEM GmbH) [19]. Measurements were performed in a controlled, quiet environment following a 5-minute seated rest period, and the cuff was selected according to the participant’s arm circumference, in accordance with standard operating procedures.

Peripheral systolic and diastolic blood pressure, as well as peripheral pulse pressure, were recorded via an Omron^®^ M5-I monitor (Omron Healthcare UK Ltd., Milton Keynes, UK) [20]. These measurements were obtained as the average of two readings taken 5 min apart, with the first taken after a minimum 5-minute rest. The participants were seated, with their right arm semiflexed and positioned at heart level, in a controlled and calm setting. Three cuff sizes were used to match the arm circumference of each participant.

#### 2.3.2. Independent Variable

The primary independent variable was sex (men/women). Sex was self-reported and was coded as a binary variable.

#### 2.3.3. Covariates

To control for potential confounding influences, both continuous and categorical covariates were included in the multivariable models:

- Continuous covariates included age (in years), body mass index (BMI, kg/m^2^), and waist circumference (cm).
- The categorical covariates included educational level, employment status, family history of myocardial infarction, family history of stroke, and smoking status.

### 2.4. Statistical Analysis

All analyses were conducted via a cleaned and harmonized dataset that integrates information from three independent sources: EVasCu, VascuNET, and ExIC-FEp. Variables were standardized in terms of naming conventions and coding schemes, and outliers were excluded prior to analysis.

Descriptive statistics are presented as medians and interquartile ranges (IQRs) for continuous variables since none of the variables followed a normal distribution according to the Shapiro–Wilk test. The homogeneity of variance was evaluated via Levene’s test. Between-group comparisons for continuous variables (men vs. women) were performed via the Mann–Whitney U test. In addition, the means and standard deviations (SDs) of the continuous parameters were reported. Categorical variables were compared via the chi-square test.

To assess whether sex-based differences in blood pressure and hemodynamic parameters persisted after controlling for potential confounders, multiple linear regression models (ordinary least squares, or OLS) were used. Each vascular parameter was modeled as the dependent variable. Sex (coded with men as the reference group) was the primary independent variable of interest. Three models were constructed:

- Model 1 (Unadjusted).
- Model 2: Age and peripheral systolic and diastolic blood pressure (this adjustment was used in all models, except when the independent variable was peripheral systolic and diastolic blood pressure and pulse pressure, to avoid overadjustment).
- Model 3 (adjusted for continuous variables): Age, BMI, and waist circumference were included as covariates.
- Model 4 (fully adjusted): This included both continuous and categorical covariates (education level, employment status, family history of CVD (myocardial infarction, stroke), and smoking status).

All analyses were performed via Python (version 3.11), which specifically employs the *scipy.stats* library for nonparametric statistical tests and the *statsmodels.formula.api* interface for the construction of linear regression models.

## 3. Results

A total of 669 participants were included in the study. The sociodemographic, clinical, and hemodynamic characteristics of the samples are presented in Table 1. With the data stratified by sex, men presented significantly greater values than women did in terms of anthropometric measurements (weight, height, BMI, and waist circumference), multiple blood pressure parameters (systolic and diastolic, central and peripheral, and pulse pressures), and certain vascular parameters, such as PWv, cardiac output, and stroke volume. Conversely, women presented higher values of AIx75 and peripheral vascular resistance.

### 3.1. Demographic, Anthropometric, and Socioeconomic Variables

Compared with women, men had significantly greater values for weight, height, BMI, and waist circumference (all *p* < 0.001). However, there were no significant differences in age (*p* = 0.113) or education level (*p* = 0.308) between the groups. Employment status was significantly different according to sex (*p* < 0.001), whereas family histories of myocardial infarction and stroke showed differing trends. Specifically, a family history of stroke was more common in women (*p* = 0.001), whereas no significant difference was found for a family history of myocardial infarction (*p* = 0.153). Smoking status also differed significantly between the sexes (*p* = 0.031).

### 3.2. Blood Pressure Parameters

Significant sex differences were observed in both central and peripheral blood pressure parameters. Men had higher central systolic blood pressure (124.0 vs. 117.0 mmHg, *p* < 0.001, d = 0.60), peripheral systolic blood pressure (126.0 vs. 113.0 mmHg, *p* < 0.001, d = 0.63), and both central and peripheral pulse pressures (*p* < 0.001 for both, d = 0.24 and 0.65, respectively). Central and peripheral diastolic pressures were also greater in men (*p* < 0.001 for both, d = 0.63 and 0.27, respectively).

**Figure 1:**
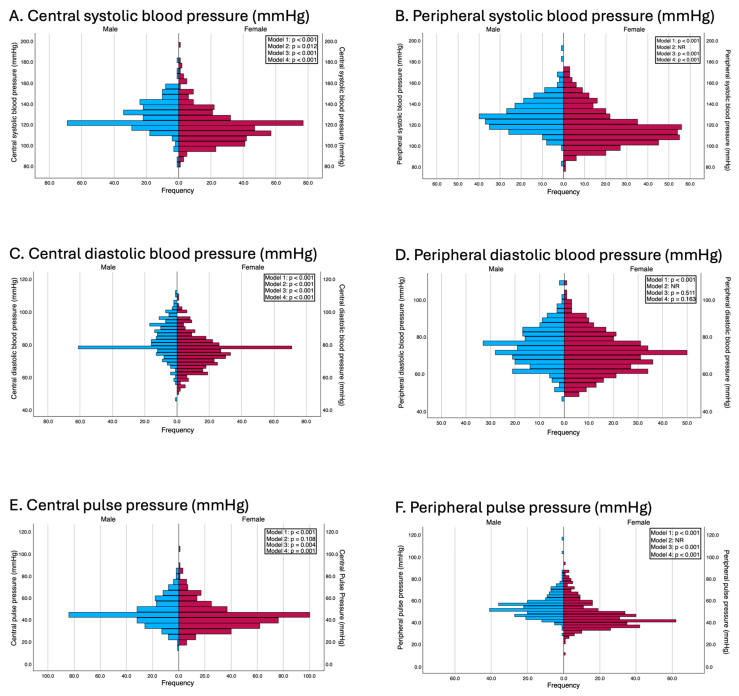
Differences between genders in terms of blood pressure parameters. Model 1: Unadjusted; Model 2: adjusted by age and peripheral systolic and diastolic blood pressure (this adjustment was used in all models except when the independent variable was peripheral systolic and diastolic blood pressures and pulse pressure, to avoid overadjustment); Model 3: adjusted by age, BMI and waist circumference; Model 4: adjusted by the variables included in model 2 and for education level, employment status, family history of myocardial infarction, family history of stroke, and smoking status.

These differences remained consistent after performing multiple linear regression analyses that adjusted for potential confounding variables. In Model 2, except for central pulse pressure (β = 1.35 mmHg; *p* = 0.108) and while excluding peripheral blood pressure parameters for which this adjustment was not made to avoid overadjustment, the systolic and central diastolic blood pressures were significantly greater in men. In Model 3, adjusted for age, body mass index (BMI), and waist circumference, men continued to present significantly greater systolic and diastolic blood pressure values. In Model 4, which was additionally adjusted for categorical variables such as educational level, employment status, family histories of myocardial infarction and stroke, and smoking status, these differences persisted. Specifically, multivariable regression models revealed that women had significantly lower peripheral systolic blood pressure (β = −9.37 mmHg; *p* < 0.001), central systolic blood pressure (β = −8.25 mmHg; *p* < 0.001), and central pulse pressure (β = −3.58 mmHg; *p* = 0.001). The results of the adjusted models are presented in Figure 1.

### 3.3. Hemodynamic Parameters

Men presented significantly greater values of PWv (7.0 vs. 6.7 m/s, *p* = 0.003, d = 0.16), cardiac output (5.0 vs. 4.4 L/min, *p* < 0.001, d = 0.70), and stroke volume (76.5 vs. 63.6 mL, *p* < 0.001, d = 0.86). Conversely, women had significantly greater AIx75 values (24.0% vs. 13.0%, *p* < 0.001, d = −0.85) and peripheral vascular resistance (1716.8 vs. 1651.8 din·s·m^2^/cm^5^, *p* = 0.002, d = −0.41).

**Figure 2:**
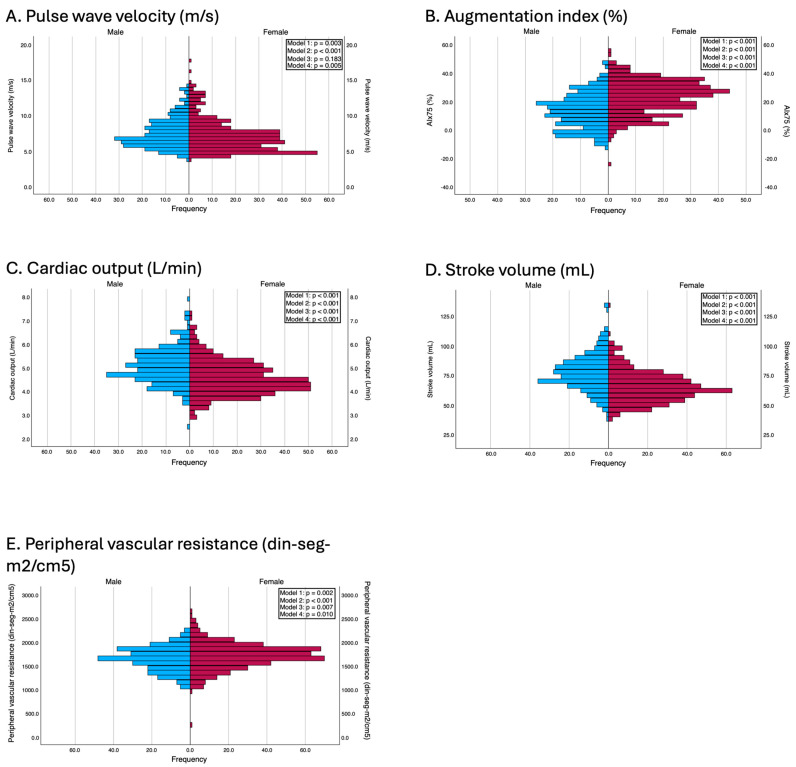
Differences between genders in terms of vascular parameters. Model 1: Unadjusted; Model 2: adjusted by age and peripheral systolic and diastolic blood pressure (this adjustment was used in all models except when the independent variable was peripheral systolic and diastolic blood pressures and pulse pressure, to avoid overadjustment); Model 3: adjusted by age, BMI, and waist circumference; Model 4: adjusted by the variables included in model 2 and education level, employment status, family history of myocardial infarction, family history of stroke, and smoking status.

These differences were also consistent after adjustment for confounders. In Model 2, all the variables maintained statistical significance, and, in Model 3, PWv lost its statistical significance (β = −0.10; *p* = 0.183). According to the fully adjusted model (Model 4), women still presented a significantly greater AIx75 (β = 11.22; *p* < 0.001) and greater peripheral vascular resistance (β = 67.05; *p* = 0.010), whereas men maintained greater cardiac output (β = −0.45; *p* < 0.001) and greater stroke volume (β = −13.27; *p* < 0.001). Notably, sex differences in PWv, which were nonsignificant in earlier models that adjusted only for age and body composition, became statistically significant after full adjustment (β = −0.17; *p* = 0.005). The results of the adjusted models are presented in Figure 2.

## 4. Discussion

This study aimed to evaluate sex-based differences in blood pressure and vascular parameters in a general adult population while adjusting for relevant demographic, anthropometric, and socioeconomic covariates. Our findings indicate that men in the cohort presented significantly greater values for central and peripheral blood pressures, PWv, cardiac output, and stroke volume, whereas the women presented higher values of AIx75 and peripheral vascular resistance. Notably, these differences persisted even after multivariable adjustment, supporting the hypothesis that biological sex is an independent determinant of vascular phenotype.

The observed sex differences in blood pressure and vascular function are consistent with previous epidemiological studies. For example, data from the Framingham Heart Study and the Asklepios Study have shown that women generally present with higher AIx75 values and lower PWv than men of the same age, which aligns with our findings [13,21]. In contrast, studies such as the Baltimore Longitudinal Study of Aging reported attenuated sex differences in PWv after adjusting for body size and blood pressure, suggesting that anthropometric factors may partially explain these differences [22]. Our results, obtained after controlling for BMI, waist circumference, and age, confirm that while some differences attenuate, others, particularly AIx75 and peripheral vascular resistance, remain significantly elevated in women, likely reflecting differences in arterial tone and vascular impedance related to hormonal, structural, or autonomic factors [10,15].

A particularly notable finding of our analysis is the shifting significance of sex differences in PWv, depending on the covariates included in the models. Initially, when adjusting for only age, BMI, and waist circumference, the difference in PWv between men and women was no longer statistically significant. This finding is pathophysiologically coherent, as PWv is highly dependent on chronological age and arterial stiffness increases with age due to the progressive fragmentation of elastin fibers, increased collagen cross-linking, and endothelial dysfunction [23,24]. These age-related vascular changes can overshadow underlying sex-related differences, especially in a relatively age-homogeneous population. However, when additional variables such as smoking status, educational level, and occupational status were incorporated, the sex difference in PWv again became statistically significant. This re-emergence may reflect the biological impact of chronic exposure to cardiovascular risk factors that differ in prevalence and effect between the sexes. For example, smoking is more prevalent in men in many populations and accelerates arterial stiffening through oxidative stress, inflammation, and endothelial injury [25]. When this effect is accounted for, intrinsic biological differences, such as hormonal protection in premenopausal women or differences in vascular smooth muscle responsiveness, become more evident [15]. Moreover, occupational and psychosocial stressors, which are differentially experienced by men and women, may influence autonomic balance and vascular tone over time [26]. Thus, the observed fluctuation in PWv significance across the models likely does not reflect a statistical artifact but rather a complex interplay between sex-specific vascular biology and differential exposure to the environmental and behavioral stressors that modulate vascular aging.

The sex-based differences observed in the vascular parameters in this study suggest the existence of different underlying physiological mechanisms between men and women. The consistently higher AIx75 and peripheral vascular resistance observed in women could reflect greater wave reflection due to smaller arterial diameters or greater vascular tone, possibly mediated by greater sympathetic activity or differences in endothelial responsiveness [12,27]. In contrast, the higher PWv, cardiac output, and stroke volume in men may be attributable to greater arterial caliber and myocardial mass, resulting in greater stroke work and faster transmission of pulse waves [14,28]. The fact that some of these differences were either more or less pronounced depending on the set of covariates included, particularly age, adiposity, and smoking, suggests that the vascular phenotype is determined by both biological sex and modifiable factors, the interactions of which may vary throughout life [29]. Additionally, hormonal factors, such as estrogen exposure, particularly pre- versus post-menopause levels, may modulate arterial compliance and vascular tone, which could explain some of the sex-specific profiles that were observed even after adjustment [15,30].

Several physiological, hormonal, and anatomical mechanisms may underlie the observed sex differences in the studied vascular parameters. Estrogens play a key role in vascular protection in women by promoting nitric oxide synthesis, modulating sympathetic tone, and improving endothelial function [31], which may partly explain their increased AIx75 values and vascular resistance. In contrast, the larger arterial diameter and greater left ventricular mass in men are associated with increased stroke volume and cardiac output [32]. A recent meta-analysis confirmed significant sex-related differences in cardiovascular structure and function, including variations in arterial distensibility, ventricular volume, and endothelial responsiveness [33]. These biological differences are complemented by behavioral factors such as physical activity, dietary habits, and smoking prevalence, which also differ between the sexes and may influence the trajectory of vascular health [34]. In addition, current clinical guidelines increasingly emphasize the need to assess cardiovascular risk according to sex, incorporating female-specific risk factors such as pre-eclampsia, premature menopause, and autoimmune diseases into clinical decision-making algorithms [35,36]. This perspective highlights the clinical importance of understanding vascular differences beyond the traditional risk factors. Mechanistic studies have also shown that both sex chromosomes and sex hormones independently contribute to vascular dimorphism, affecting the endothelial cell phenotype, extracellular matrix remodeling, and inflammatory regulation [37,38]. These molecular and physiological insights collectively underscore the importance of accounting for sex differences in the assessment and treatment of vascular health.

This study has several strengths, including the use of harmonized data from multiple population-based cohorts, standardized measurement protocols with validated devices (Mobil-O-Graph and Omron^®^), and robust statistical models accounting for a wide range of potential confounders [5,39,40]. However, several limitations should be acknowledged. First, the cross-sectional design limits our ability to infer causality or the temporal progression of vascular changes [41]. Second, although sex was coded as binary, this approach does not capture the full spectrum of sex-related influences on health, which may involve gender identity, hormonal status, or sociocultural dimensions [42]. Third, some residual confounding factors may persist despite extensive adjustment, particularly for unmeasured variables such as physical activity, dietary patterns, or hormone therapy use, all of which are known to influence vascular health [43,44]. Finally, the study population was not ethnically diverse, which may limit the generalizability of the findings to broader populations with different genetic, environmental, or cultural backgrounds [45].

## 5. Conclusions

In conclusion, our study revealed significant sex differences in blood pressure and vascular parameters: women have greater wave reflection and vascular resistance, while men have greater arterial stiffness and cardiac output. These differences persist even after accounting for key confounding variables, suggesting that sex is an independent determinant of vascular phenotype. These findings underscore the importance of incorporating sex-specific reference values and treatment strategies in clinical practice. Future research should continue to explore the biological and social determinants of vascular health across the lifespan and assess whether individualized cardiovascular prevention strategies based on sex can improve outcomes.

## Figures and Tables

**Table 1:** Characteristics of the study sample.

Variable		Men	Women	Cohen’s d	*p*-Value
		*n* = 260	*n* = 409		
**Demographic, anthropometric, and socioeconomic variables**					
Age (years)	Median (IQR)	49.00 (38.00–61.00)	48.00 (36.00–58.00)	0.13 (−0.03–0.28)	0.113
	Mean (SD)	49.61 (16.80)	47.39 (17.83)		
Weight (kg)	Median (IQR)	80.53 (73.29–89.54)	66.45 (56.65–74.05)	1.13 (0.96–1.30)	<0.001
	Mean (SD)	82.02 (14.25)	66.28 (13.75)		
Height (cm)	Median (IQR)	174.05 (168.74–178.38)	161.05 (156.15–165.40)	1.68 (1.50–1.86)	<0.001
	Mean (SD)	173.76 (8.12)	160.66 (7.58)		
BMI (kg/m^2^)	Median (IQR)	26.71 (24.20–29.40)	24.84 (21.68–23.03)	0.30 (0.15–0.46)	<0.001
	Mean (SD)	27.18 (4.44)	25.70 (5.19)		
Waist Circumference (cm)	Median (IQR)	92.55 (84.19–102.59)	81.35 (70.80–90.55)	0.86 (0.70–1.02)	<0.001
	Mean (SD)	94.14 (14.45)	82.00 (13.94)		
**Education Level**					
Cannot read or write	*n* (%)	0 (0.0)	2 (100.0)	-	0.308
No education	*n* (%)	10 (32.3)	21 (67.7)		
Elementary education	*n* (%)	24 (53.3)	21 (46.7)		
School graduate	*n* (%)	46 (37.4)	77 (62.6)		
High school education	*n* (%)	74 (38.9)	116 (61.1)		
University degree completed		106 (38.1)	172 (61.9)		
**Employment Status (*n*, %)**					
Householder	*n* (%)	3 (6.4)	44 (93.6)	-	<0.001
Student	*n* (%)	23 (28.8)	57 (71.2)		
Unemployed	*n* (%)	56 (56.6)	43 (43.4)		
Employee	*n* (%)	128 (35.7)	231 (64.3)		
Self-employed worker or business owner	*n* (%)	50 (59.5)	34 (40.5)		
**Family History of myocardial infarction**					
Yes	*n* (%)	74 (43.8)	95 (56.2)	-	0.153
No	*n* (%)	186 (37.2)	314 (62.8)		
**Family History of Stroke**					
Yes	*n* (%)	31 (25.6)	90 (74.4)	-	0.001
No	*n* (%)	229 (41.8)	319 (58.2)		
**Smoking status**					
Yes	*n* (%)	34 (37.0)	58 (63.0)	-	0.031
Ex-smoker, 0–1 year	*n* (%)	8 (38.1)	13 (61.9)		
Ex-smoker, 1–5 years	*n* (%)	12 (48.0)	13 (52.0)		
Ex-smoker, >5 years	*n* (%)	65 (50.4)	64 (49.6)		
Non-smoker	*n* (%)	141 (35.1)	261 (64.9)		
**Blood pressure parameters**					
Central systolic blood pressure (mmHg)	Median (IQR)	124.00 (120.00–136.00)	117.00 (107.00–125.00)	0.60 (0.44–0.76)	<0.001
	Mean (SD)	127.27 (13.90)	118.09 (16.24)		
Peripheral systolic blood pressure (mmHg)	Median (IQR)	126.00 (117.00–136.62)	113.00 (105.00–126.00)	0.63 (0.47–0.79)	<0.001
	Mean (SD)	127.48 (14.86)	116.93 (17.70)		
Central diastolic blood pressure (mmHg)	Median (IQR)	79.50 (77.00–89.00)	76.00 (68.00–80.00)	0.63 (0.47–0.78)	<0.001
	Mean (SD)	81.58 (10.22)	75.25 (10.05)		
Peripheral diastolic blood pressure (mmHg)	Median (IQR)	73.00 (66.00–80.50)	70.00 (63.00–77.00)	0.27 (0.12–0.43)	<0.001
	Mean (SD)	73.53 (10.50)	70.59 (10.87)		
Central pulse pressure (mmHg)	Median (IQR)	42.00 (40.00–51.00)	42.00 (35.00–48.00)	0.24 (0.08–0.39)	<0.001
	Mean (SD)	45.57 (11.64)	42.73 (12.17)		
Peripheral pulse pressure (mmHg)	Median (IQR)	52.75 (47.00–58.25)	44.00 (38.00–52.00)	0.65 (0.50–0.81)	<0.001
	Mean (SD)	53.95 (10.47)	46.38 (12.21)		
**Vascular parameters**					
Pulse wave velocity (m/s)	Median (IQR)	7.00 (5.90–8.90)	6.70 (5.20–8.10)	0.16 (0.00–0.32)	0.003
	Mean (SD)	7.54 (2.15)	7.17 (2.41)		
AIx75 (%)	Median (IQR)	13.00 (4.00–21.00)	24.00 (16.00–32.00)	−0.85 (−1.01–−0.69)	<0.001
	Mean (SD)	13.20 (12.17)	23.29 (11.74)		
Cardiac output (L/min)	Median (IQR)	5.00 (4.50–5.50)	4.40 (4.00–5.00)	0.70 (0.54–0.86)	<0.001
	Mean (SD)	5.01 (0.76)	4.50 (0.71)		
Stroke volume (mL)	Median (IQR)	76.50 (67.70–85.85)	63.60 (56.10–72.30)	0.86 (0.70–1.02)	<0.001
	Mean (SD)	77.27 (15.49)	65.24 (13.02)		
Peripheral vascular resistance (din-seg-m^2^/cm^5^)	Median (IQR)	1651.85 (1451.50–1834.00)	1716.80 (1570.20–1869.90)	−0.41 (−0.57–−0.25)	0.002
	Mean (SD)	1641.22 (261.03)	1707.88 (10.05)		

IQR: interquartile range; SD: standard deviation; Aix75: augmentation index. Positive values in Cohen’s d are in favor of men, and negative values in cohen’s d are in favor of women.

## Data Availability

Data is contained within the article.

## Abbreviations

The following abbreviations are used in this manuscript:

AIx75	Augmentation index, normalized to 75 beats per minute
BMI	Body mass index
CVD	Cardiovascular disease
IQR	Interquartile range
OLS	Ordinary least squares
PWv	Pulse wave velocity
